# Pattern of adverse events induced by aflibercept and ranibizumab: A nationwide spontaneous adverse event reporting database, 2007–2016

**DOI:** 10.1097/MD.0000000000016785

**Published:** 2019-08-16

**Authors:** Dongmun Ha, So-Ra Choi, Yongmin Kwon, Han-Heui Park, Ju-Young Shin

**Affiliations:** School of Pharmacy, Sungkyunkwan University, 2066 Seobu-ro, Jangan-gu, Suwon, Gyeonggi-do, South Korea.

**Keywords:** age-related macular degeneration, data mining, drug-AE, KAERS database, signal detection

## Abstract

Data regarding the safety of anti-vascular endothelial growth factor (anti-VEGF) treatment is limited.

To compare the adverse events (AEs) induced by aflibercept and ranibizumab using a spontaneous reporting system and determine the signals.

We used data from the Korea Institute of Drug Safety & Risk Management-Korea Adverse Event Reporting System Database (KIDS-KD), collected between 2007 and 2016. Differences in patient demographics, report type, reporter, causality, and serious-AEs between aflibercept and ranibizumab were compared. Metrics including proportional reporting ratio (PRR), reporting odds ratio (ROR), and information component (IC), were used to compare signals with the AEs on the drug labels in the United States of America and Korea. Logistic regression analysis was performed to identify AEs that are more likely to occur with drug use.

A total of 32 aflibercept and 103 ranibizumab cases of AEs were identified. The proportion of AEs that were reported voluntarily was higher with aflibercept (50.5%) use than ranibizumab (4.9%), whereas the AEs reported by post-marketing surveillance were higher with ranibizumab (46.6%) use than aflibercept (31.3%). The percentage of AEs in patients >60 years old, reports by consumers, and the ratio of SAEs to AEs associated with aflibercept (84. %, 9.4%, and 75.0%, respectively) were higher than those of ranibizumab (77.7%, 1.9%, and 19.4%, respectively). The number of newly detected AEs after aflibercept and ranibizumab treatment was 3 and 8, respectively. Among these, conjunctivitis and medicine ineffective were not included on the aflibercept and ranibizumab labels, respectively. Endophthalmitis (OR 6.96, 95% CI 2.74–17.73) was more likely to be reported in patients with aflibercept than in patients without aflibercept, whereas medicine ineffective (OR 18.49, 95% CI 2.39–143.29) and retinal disorder (OR 7.03, 95% CI 1.60–30.96) were more likely to be reported in patients with ranibizumab than in patients without ranibizumab.

New signals have been identified for aflibercept and ranibizumab. Further research is necessary to evaluate the causality of AEs that were detected as signals in this study.

## Abstract 2

Supplemental Digital Content is available in the text

## 1 Introduction

Injections of aflibercept and ranibizumab, which are anti-vascular endothelial growth factors (anti-VEGFs), have been used in the treatment of various diseases of the retina, including diabetic macular edema (DME), diabetic retinopathy (DR), exudative age-related macular degeneration (AMD), retinal vein occlusions (RVO), myopic choroidal neovascularization, and radiation retinopathy.^[1,2,3,4,5,6]^ Since the mid-2000s, intraocular injection of agents that inhibit VEGF, which is a critical mediator of physiological angiogenesis and pathological angiogenesis,^[7]^ has become the mainstay of treatment for these diseases.^[8]^ Currently, aflibercept (approved March 20th, 2013) and ranibizumab (approved July 27th, 2007) are marketed for the treatment of ocular diseases in South Korea.^[9]^ Bevacizumab (approved September 12th, 2007), another anti-VEGF, is only used off-label for ophthalmic purposes. However, despite the use of anti-VEGF for the treatment of ophthalmic diseases, its safety has not been fully evaluated.

Recently, 2472 cases of anti-VEGF drug-related AEs were identified in the areas of oncology and ophthalmology, by analyzing a spontaneous reporting database in Italy.^[10]^ However, the main limitation of this study was the inability to detect new AEs that were not included on the labels of anti-VEGF drugs, considering that the analyses were based on the AEs indicated on the labels. Nevertheless, no pharmacovigilance studies have employed data mining of an adverse event (AE) reporting database by matching specific AEs with the use of anti-VEGF agents in South Korea. Therefore, the objective of this study was to compare the AEs of anti-VEGF agents by using a spontaneous reporting system, to determine signals of anti-VEGF agents, and to compare the data collected to the drug labels of anti-VEGF therapies marketed in the United States and Korea.

## 2 Methods

### 2.1 Database and study drugs

We employed spontaneous AE reports from the Korea Adverse Events Reporting System (KAERS) database. The KAERS system was developed by the Korean Institute of Drug Safety & Risk Management (KIDS) in 2012 to promote the reporting of AEs and management of AE reports. All spontaneous AE reports have been reported in the KAERS from 1988, which was the inaugural year of the spontaneous AE reporting system in Korea.^[11]^ All variables employed in this study were categorized in accordance with the guidelines for the KIDS KAERS Database (KIDS-KD), and used by research, medical, and, public institutions. We collected all the information (reporting year, sex, age, reporting type, and source, drug use, AEs, and causality evaluation) contained in the KIDS-KD. A search for AEs following drug use was conducted, and only the AEs that were categorized by the World Health Organization-Uppsala Monitoring Center (WHO-UMC) causality assessment system as “certain,” “probable,” or “possible” were used in this study. Data with missing ATC/AE codes, age, and sex were excluded from the analysis. In addition, only the initial reports for the suspected drugs were used in our analysis. Regarding search and classification codes, the ATC code was employed for drugs, whereas the World Health Organization-Adverse Reactions Terminology (WHO-ART) version 092 was employed for the AEs. We reviewed AEs reported between July 1, 2007, and December 31, 2016, and selected anti-VEGF agents (aflibercept and ranibizumab) for retinal diseases, such as exudative AMD and RVO. Aflibercept or ranibizumab that are marketed in South Korea are the study drugs and used the other drugs as the comparator drugs. For example, when the study drug was aflibercept, the comparator drug was ranibizumab.

### 2.2 Aflibercept and ranibizumab: AE reports and AE pairs

AEs were considered as codes for the side effects, so they were extracted as PT (preferred term) codes. Because this study consisted of the analysis of all the AEs that occurred for the study drugs, drug-AE pairs were created by using one-to-one correspondence between the drugs and the AEs, and so we compared the characteristics of AEs by computing their frequency and percentage (%).

### 2.3 Comparison of AE status of aflibercept and ranibizumab

Between July 2007 and December 2016, the annual proportion and annual rate of increase in the frequency of reported AEs in response to aflibercept and ranibizumab use was calculated. In addition, the ratio of serious adverse events (SAE) among all AEs was calculated for each treatment.

### 2.4 Demographic characteristics

Information on sex and age was extracted. The patients were categorized into 2 subgroups according to sex: male and female. The ages of the patients were reclassified into 4 subgroups based on the female menstrual period, fertility period, and menopausal period: younger than 20 years; between 20 and 39 years; between 40 and 59 years; and older than 60 years. The AE-pairs extracted from the demographic characteristics were classified into 2 groups: aflibercept and ranibizumab.

### 2.5 Report type and report source by profession

The data were analyzed by report type (spontaneous, research, article, and others) and source of report by profession (doctor, pharmacist, nurse, customer, other, and unknown).

### 2.6 Causality assessment and SAEs

The causality evaluation of AE-pairs related to aflibercept and ranibizumab was performed based on the WHO-UMC causality assessment system. The evidence was categorized into three levels (certain, probable, and possible). “Certain” was defined as either,

(1) an event or laboratory test abnormality with a reasonable temporal relationship to the drug intake,
(2) a definite pharmacological or phenomenological event (e.g., “grey baby syndrome,” chloramphenicol, or anaphylaxis immediately after the administration of a drug that had been administered previously),
(3) events that cannot be explained by the disease or another drug, or
(4) response to withdrawal plausible and re-challenge satisfactory if necessary.

“Probable,” was defined as either the event does not appear to be due to the disease or other drugs or the response to withdrawal is clinically reasonable and a re-test is not required. “Possible,” was defined as the event that could be explained by the disease or other drug and there is no information available on the consequences after drug withdrawal. Two types of SAEs, hospital and other (including death), were compared between the 2 groups (aflibercept and ranibizumab).

### 2.7 Signal indices by data mining

After all the combinations of the drug-adverse effects were obtained, the data mining method was used to analyze three types of signal detection information. Data mining is defined as a process in which beneficial information is discovered from a large set of data; it is a technology which finds not only expected results, but also new and unexpected information. The techniques of data mining are used to search for unexpected associations or hidden patterns in a wide range of databases via computerized algorithms.^[12]^ It is also referred to as a technique that correlates data to generate valuable information that is applied to decision-making processes. A quantitative vs a qualitative method was used to calculate the imbalance report fraction and analyze the results in signal detection. Imbalance measurement is a type of statistical technique that detect the signals of AEs and is a fundamental analysis method used in pharmacovigilance.^[13]^ A 2 × 2 table was constructed, in which each column contained information related to the research drug, all other drugs, specific AEs, and all other AEs.^[14]^ In other words, if the research drug had a specific AE it would fall into column A, if the research drug had all other AEs it would be in column B, if all other drugs had a specific AE it would be in column C, and if all other drugs had all other AEs it would fall into column D.

To detect signals, the proportional reporting ratio (PRR), reporting odds ratio (ROR), and information component (IC) of Bayesian confidence propagation neural network (BCPNN) were computed.^[15,16,17]^ PRR relates to the fraction of specific AEs in specific drugs divided by the fraction of specific AEs in all other drugs, and its corresponding formula is (2)/(4). The signal judgment criteria were PRR ≥2, chi-square ≥4, and the number of cases with AEs ≥3.^[18]^ ROR was defined as the odds of a specific AE outcome from a specific drug exposure divided by the odds of a specific AE outcome from exposure to all other drugs. The formula is (A/C) / (B/D), and the signal judgment criterion comprised of a ROR ≥2, chi-squared ≥4, and the number of cases of AEs ≥3.^[19]^ The IC is the logarithmic value of the probability of using a certain drug multiplied by the probability of the occurrence of a specific AE, if the use of that drug and the occurrence of the particular AE are independent of each other. The formula for the calculation of IC is 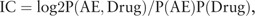 and the criterion is when the lower limit of the 95% confidence interval is higher than 0.^[12]^ In this study, AEs that satisfied all three criteria (PRR, ROR, and IC) were defined as signals.^[20]^ The signals detected from the KAERS database were compared with the Korean and US drug label information. The US label was obtained from the Daily Med website.^[21]^ We first confirmed whether a signal was a new AE based on its license approval; categorized signals not listed on the drug label were classified as unexpected signals.

### 2.8 Statistical analysis

To verify that the dates of approval for both anti-VEGF agents were different from each other, and to confirm changes in both frequency and trends in AE reports over time, a comparative analysis was performed for each year based on the reported information. We calculated the frequency and percentage (%) for each categorical variable. Statistical analysis was performed in accordance with the characteristics of each variable defined above. *P* values of <.05 by Mantel-Haenszel chi-squared test were considered statistically significant. Logistic regression analysis was performed by controlling variables of sex and age to compute odds ratios (ORs) and 95% confidence intervals (CIs) for the ROR of AEs due to drug use. All statistical analyses were performed using SAS 9.4 (SAS Institute Inc., Cary, NC) and Excel 2010 (Microsoft Corp., Redmond, WA), and the detected signals were compared with the drug label information approved in Korea and by the USA Food and Drug Administration (FDA). The study protocol was approved by the Institutional Review Board of Sungkyunkwan University (approval number: 2018–01–026).

## 3 Results

Between July 1, 2007, and December 31, 2016, the total number of AE reported due to ranibizumab (103) use was greater than that of aflibercept (32). According to the reports of AEs resulting from anti-VEGF agents, AEs resulting from aflibercept (71.9%) and ranibizumab (62.1%) were reported more frequently by men (Table 1). AEs induced by anti-VEGF agents were most commonly reported in patients >60 years old, of which the proportion of aflibercept (84.4%)-related AEs was higher than that related to ranibizumab (77.7%). The number and percentage of reports by post-marketing surveillance was higher with ranibizumab (48 cases, 46.6%) use than aflibercept (10, 31.3%) (Table 1). The percentage of AEs reported by consumers and the ratio of SAEs to AEs with aflibercept use (9.4% and 75.0%, respectively) were higher than those of ranibizumab (1.9% and 19.4%, respectively) (Table 1).

**Table 1:**
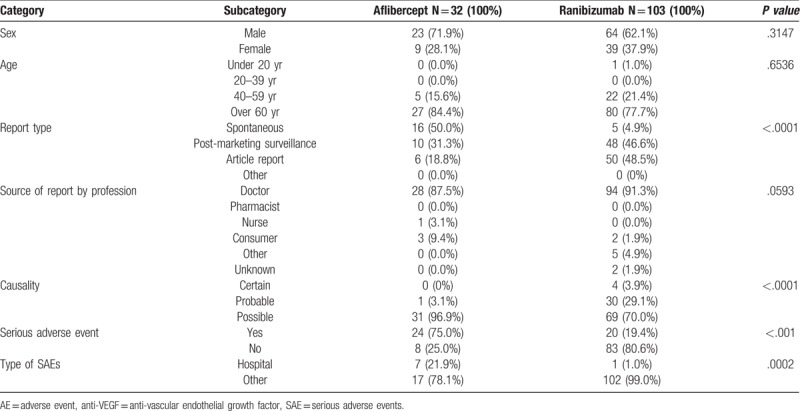
Characterization of adverse events (AEs), causality, and serious adverse events (SAEs) associated with the use of anti-VEGF agents between July 2007 and December 2016.

**Figure 1:**
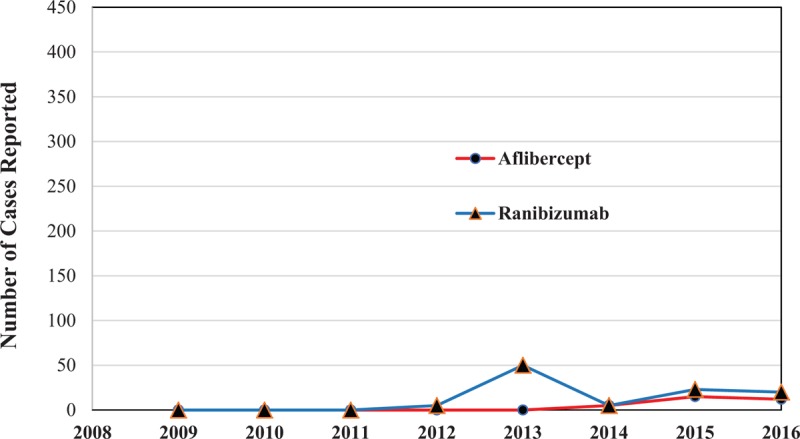
Number of reported cases of adverse events (AEs) associated with the use of anti-vascular endothelial growth factor (anti-VEGF) agents, by year. VEGF = vascular endothelial growth factor.

**Figure 2:**
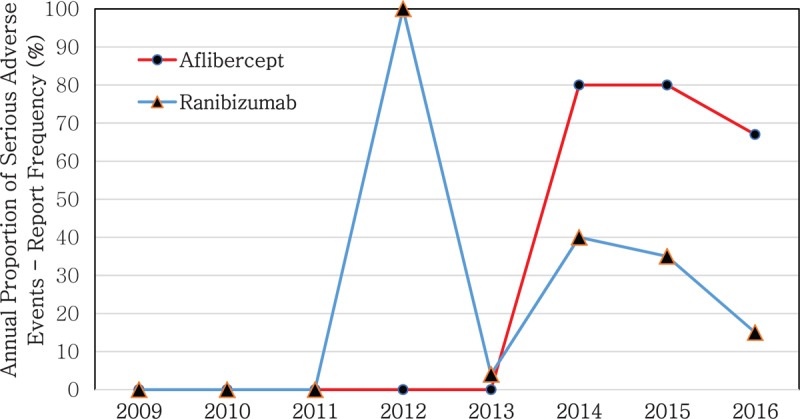
Reported frequency of serious adverse events (SAEs) associated with the use of anti-vascular endothelial growth factor (anti-VEGF) agents, by year. VEGF = vascular endothelial growth factor.

The annual frequency and proportion of AE induced by the 2 therapeutic agents increased between 2011 and 2016 (Fig. 1). The ratio of ranibizumab-induced SAEs to AEs, was higher than the ratio of aflibercept-induced SAEs to AEs. More than 60% of the reported cases of aflibercept AEs diagnosed after 2014 were SAEs, whereas ranibizumab had the highest ratio of SAEs to AEs in 2013 (100.0%). The number of cases of AE due to ranibizumab also increased by more than 10 times in 2013 compared to 2012 (Fig. 2).

For aflibercept, 3 types of AEs, namely endophthalmitis, conjunctivitis, and muscae volitantes, were confirmed to be signal information. Among these, conjunctivitis was not listed on the label (Table 2). For ranibizumab, eight types of AEs, such as retinal disorder, medicine ineffective, endophthalmitis, retinal detachment, retinal hemorrhage, vision abnormal, conjunctivitis, and muscae volitantes, were found to be signal information. Among these, medicine ineffective was not listed on the label (Table 3).

**Table 2:**
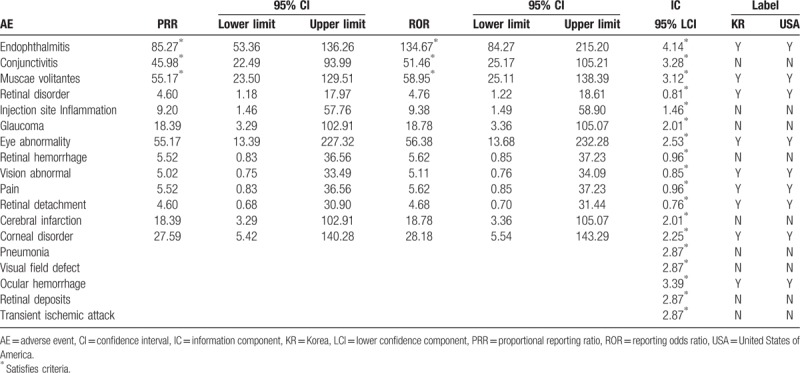
Comparison of detected signals and World Health Organization-Adverse Reaction Terminology (Preferred Terms) labeling of aflibercept-associated adverse events (AEs) from July 2007 to December 2016 in South Korea.

**Table 3:**
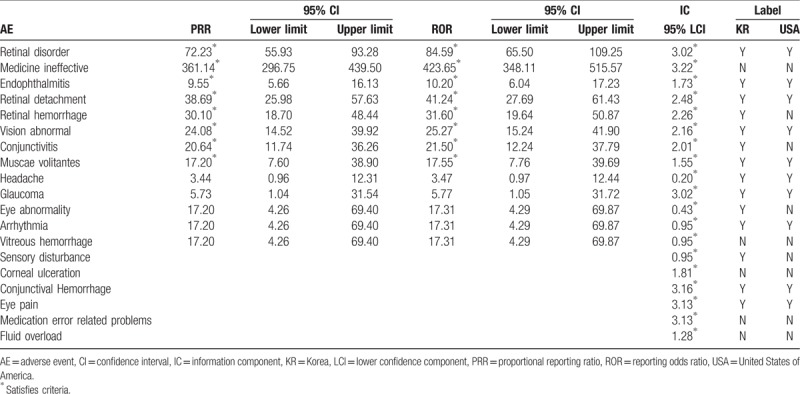
Comparison of detected signals and World Health Organization-Adverse Reaction Terminology (Preferred Terms) labeling of ranibizumab associated adverse events (AEs) from July 2007 to December 2016 in South Korea.

The logistic regression analysis results showed that patients treated with aflibercept were 6.96 times significantly more likely to present endophthalmitis (OR 6.96, 95% CI 2.74–17.73) than those treated with other drugs. Compared to patients treated with other drugs, patients treated with aflibercept were also more likely to present AEs such as muscae volitantes (OR 4.93, 95% CI 0.75–32.65), conjunctivitis (OR 3.03, 95% CI 0.80–11.52), and eye abnormality (OR 2.72, 95% CI 0.16–46.68); however, the difference was not statistically significant. Patients treated with ranibizumab were 18.49 and 7.03 times significantly more likely to present medicine ineffective (OR 18.49, 95% CI 2.39–143.29) and retinal disorder (OR 7.03, 95% CI 1.60–30.96), respectively, than those treated with other drugs. Patients treated with ranibizumab were also more likely to present AEs such as retinal detachment (OR 2.72, 95% CI 0.57–13.07) and retinal hemorrhage (OR 2.02, 95% CI 0.40–10.14) than those treated with other drugs, but the difference was not statistically significant (Table 4).

**Table 4:**
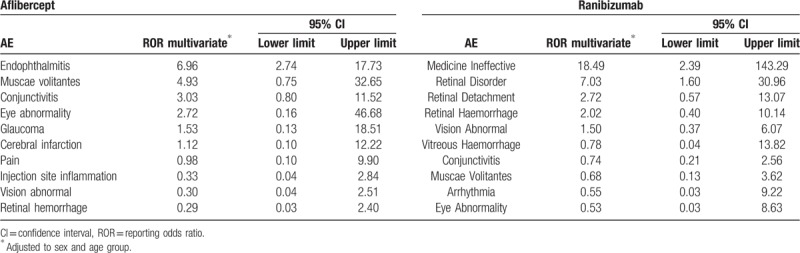
Logistic regression model adjusted to sex and age group for adverse events induced by aflibercept and ranibizumab from July 2007 to December 2016 in South Korea.

## 4 Discussion

Our study describes AEs induced by anti-VEFG treatment using data-mining to identify new AEs not defined on drug labels approved by the USA FDA or regulatory agencies in Korea. New AEs caused by aflibercept (3) and ranibizumab (8) were identified, of which, conjunctivitis and medicine ineffective were not included on the drug labels of aflibercept and ranibizumab, respectively.

The proportion of AEs caused by anti-VEGF agents was different for each treatment type. Approximately 50% of the aflibercept AEs was reported through voluntary reporting, whereas approximately 31.3% was reported through post-marketing surveillance. The review period post approval, which was 4 or 6 years after drug approval,^[22]^ has not yet expired, and the proportion of voluntary reporting for aflibercept may be higher than that reported through post-marketing surveillance. Approximately 46.6% of ranibizumab AEs was reported through post-marketing surveillance, and approximately 48.5% through literature.

The most frequently reported AEs varied between the anti-VEGF agents. The most frequently reported AE caused by aflibercept was endophthalmitis (37%), followed by conjunctivitis, vitreous floaters, retinal disease, and ocular hemorrhage. The most frequently reported AEs caused by ranibizumab were retinal diseases and medicine ineffective, which accounted for 14.8% each, followed by conjunctival hemorrhage and acanthosis. According to Heier et al,^[23]^ AEs such as conjunctival hemorrhage, aching, retinal hemorrhage, decreased visual acuity, retinal detachment, and increased intraocular pressure were reported with both aflibercept and ranibizumab treatment.^[23]^ However, muscae volitantes with aflibercept use and medicine ineffective due to ranibizumab were newly identified AEs in this study.

Conjunctivitis and medicine ineffective were not included on the drug labels of aflibercept and ranibizumab, respectively. Although medicine ineffective represented 14.8% of the AEs caused by ranibizumab, there was almost no mention of this in the research studies or the information was not included on the label. Approximately 75.0% of the AEs caused by aflibercept were SAEs and 19.4% of the AEs caused by ranibizumab were described as SAEs. Since a high frequency of the AEs caused by ranibizumab was reported as medicine ineffective, most of these AEs were classified as SAEs. According a study by Bakall et al,^[24]^ patients who were either resistant to ranibizumab or had a recurrence of AMD showed a therapeutic response to aflibercept injection.^[24]^ There was also the possibility that signal information of ranibizumab, such as medicine ineffective, may have affected the results of this study. Another study reported that aflibercept was more effective than ranibizumab in a patient who experienced serous pigment epithelial detachment.^[25]^ It was assumed that the broad ligand-binding spectrum of aflibercept may have affected the outcomes of this research.^[25]^ Therefore, additional studies are required to determine whether the broad ligand-binding spectrum influenced the high frequency of medicine ineffective reported as an AE associated with ranibizumab. Conversely, the signal information for bevacizumab, which is currently used off-label for the treatment of ophthalmic diseases in Korea, identified 6 AEs (anorexia, cachexia, neuropathy peripheral, hypertension, palmar-plantar erythrodysesthesia, and mucositis). Additionally, all signal information related to bevacizumab could be confirmed on the label (Appendix 1). It is assumed that this probably occurs because bevacizumab is a cancer drug that is prescribed to a large fraction of the population. Given the increase in the sample population, the number of treated patients also increased, as did the probability of an AE occurring. Therefore, it is likely that the labeling of signal information for aflibercept and ranibizumab through research might have occurred more quickly.

Our study had remarkable strengths. We generated representative results that included all reports from all groups, including the data reported by pharmaceutical companies in a nationwide AE report database in South Korea. To the best of our knowledge, this is the first study identifying and comparing AEs of anti-VEGF treatment agents in South Korea. Furthermore, we verified, using recent databases, the current status of completeness of AE reporting systems.

Nevertheless, our findings have the following limitations. First, various AEs were underreported in this study. Given that voluntary reporting does not include the entire patient population prescribed the drug, it was difficult to define the incidence of the disease, even though a high number of AEs were reported. Although a significant amount of highly reported AEs was observed, such information should not define the causal relationship between the specific treatment and the AE, but can be used as evidence for causality. Second, the number of cases of AEs in response to treatment with anti-VEGF agents was not large enough to be considered viable. Since the actual number of reports is small, each proportion can be overestimated. Thus, the results of this study should be carefully considered when generalizing the findings.

In conclusion, new AE signals were detected in patients treated with anti-VEGF agents. Therefore, it is necessary to evaluate the causality of the AEs detected as signals in this study to ensure patient safety in the future.

## Author contributions

**Conceptualization:** Dongmun Ha, So-Ra Choi.

**Data curation:** So-Ra Choi, Yongmin Kwon, Han-Heui Park.

**Formal analysis:** So-Ra Choi, Yongmin Kwon, Han-Heui Park.

**Methodology:** Dongmun Ha, So-Ra Choi.

**Project administration:** Ju-Young Shin.

**Resources:** Yongmin Kwon.

**Supervision:** Ju-Young Shin.

**Validation:** So-Ra Choi.

**Writing – original draft:** Dongmun Ha.

**Writing – review & editing:** Ju-Young Shin.

Dongmun Ha orcid: 0000-0002-1573-8265.

## Supplementary Material

Supplemental Digital Content
