# Supplementary material for: Pattern of adverse events induced by aflibercept and ranibizumab: A nationwide spontaneous adverse event reporting database, 2007–2016
Source: Medicine (Baltimore). 2019 Aug 16;98(33):e16785. doi: 10.1097/MD.0000000000016785 (PMC6831246; doi:10.1097/MD.0000000000016785)
Supplement: Supplemental Digital Content [file medi-98-e16785-s001.docx]

**Appendix.** Comparison of detected signals and World Health Organization-Adverse Reaction Terminology (Preferred Terms) labeling of bevacizumab-associated adverse events (AEs) from July 2007 to December 2016 in South Korea

| AE | PRR | 95% CI | | ROR | 95% CI | | IC  95% LCI | Label | |
| --- | --- | --- | --- | --- | --- | --- | --- | --- | --- |
|  |  | Lower limit | Upper limit |  | Lower limit | Upper limit |  | KR | USA |
| Anorexia | 2.12* | 1.82 | 2.46 | 2.19* | 1.88 | 2.54 | 0.07* | Y | Y |
| Cachexia | 28.50* | 26.48 | 30.68 | 29.64* | 27.54 | 31.91 | 0.51* | Y | N |
| Neuropathy Peripheral | 3.41* | 2.73 | 4.25 | 3.45* | 2.77 | 4.31 | -0.06 | Y | Y |
| Hypertension | 2.94* | 2.30 | 3.77 | 2.98* | 2.32 | 3.82 | -0.17 | Y | Y |
| Palmar-Plantar Erythrodysesthesia | 2.97* | 2.18 | 4.05 | 2.99* | 2.20 | 4.08 | -0.36 | Y | Y |
| Mucositis Nos | 3.72* | 2.67 | 5.17 | 3.74* | 2.69 | 5.20 | -0.43 | Y | Y |

Abbreviations: PRR, proportional reporting ratio; ROR, reporting odds ratio; IC, information component, CI, confidence interval; LCI, lower confidence component; KR, Korea; USA, United States of America

* Satisfies criteria
